# Comparison of the healthcare system of Chile and Brazil: strengths, inefficiencies, and expenditures

**DOI:** 10.1186/s12962-022-00405-9

**Published:** 2022-12-16

**Authors:** Arcadio A. Cerda, Leidy Y. García, Jennifer Rivera-Arroyo, Andrés Riquelme, Joao Paulo Teixeira, Mihajlo Jakovljevic

**Affiliations:** 1 Faculty of Economics and Business, University of Talca, Talca, Chile; 2 Facultad de Administración y Negocios, Universidad Autónoma de Chile, Talca, Chile; 3 Faculty of Engineering of the University of Porto (FEUP), Porto, Portugal; 4 Polytechnic Institute of Bragança, Bragança, Portugal; 5 Institute of Advanced Manufacturing Technologies, Peter the Great St. Petersburg Polytechnic University, St. Petersburg, Russia; 6 Institute of Comparative Economic Studies, Faculty of Economics, Hosei University, Tokyo, Japan; 7 Department of Global Health Economics and Policy, University of Kragujevac, Kragujevac, Serbia

**Keywords:** Health system, Allocation, Expenditure, Sustainability

## Abstract

**Background:**

Governments in Latin America are constantly facing the problem of managing scarce resources to satisfy alternative needs, such as housing, education, food, and healthcare security. Those needs, combined with increasing crime levels, require financial resources to be solved.

**Objective:**

The objective of this review was to characterizar the health system and health expenditure of a large country (Brazil) and a small country (Chile) and identify some of the challenges these two countries face in improving the health services of their population.

**Methods:**

A literature review was conducted by searching journals, databases, and other electronic resources to identify articles and research publications describing health systems in Brazil and Chile.

**Results:**

The review showed that the economic restriction and the economic cycle have an impact on the funding of the public health system. This result was true for the Brazilian health system after 2016, despite the change to a unique health system one decade earlier. In the case of Chile, there are different positions about which one is the best health system: a dual public and private or just public one. As a result, a referendum on September 4, 2022, of a new constitution, which incorporated a unique health system, was rejected. At the same time, the Government ended the copayment in the public health system in September 2022, excluding illnesses referred to the private sector. Another issue detected was the fragility of the public and private sector coverage due to the lack of funding.

**Conclusions:**

The health care system in Chile and Brazil has improved in the last decades. However, the public healthcare systems still need additional funding and efficiency improvement to respond to the growing health requirements needed from the population.

## Introduction

Governments in Latin America are constantly facing the problem of managing scarce resources to satisfy alternative needs, such as housing, education, food, and healthcare security. Those needs, combined with increasing crime levels, require financial resources to be solved. Given the above, the provision of resources to support the different health systems falls not only on the Government’s responsibility but also on individuals through the acquisition of insurance or on mixed financing systems for social security and health care [1]. Atun et al. [2] mention that governments in Latin American countries are making reforms to improve the health system to reduce inequality based on access and expansion of universal health coverage using the principles of equity and solidarity. The increase of benefits for the population becomes even more complex when countries face low economic growth rates, which limits tax collection to meet society’s multiple and increasing needs.

Despite this, some countries, like Chile, still propose to have only a unique, universal public healthcare system. In other words, some local political sectors intend to move about 3 million people from the private healthcare system to public health. However, the public system still has over 2 million consultations with specialists and over one-third of a million surgical operations with a waiting time of almost 2 years [3]. Other countries like Brazil, which has a mixed health care system, is supported by central, regional, and local Government, which always have excess demands for health care services. The literature recognizes three standard health systems [4, 5]: (a) A dual health system: the State covers the poor while most individuals with better financial conditions are directed to private health insurance; (b) A universal health system model: public and universal system, but that coexists with private insurance used, mainly, by the middle class. (c) A plural health system model: public and private supplier companies in competition are part, with a mechanism that includes the poorest through insurance.

This manuscript aims to characterize and compare the health care system of Chile (dual Model), a relatively small country with a population of 18 million, and Brazil (a universal health system model), a relatively large country with a population of 212.6 million. The method used to conduct this research is by literature review and data analysis using multiple sources of information.

## Characterization of the health system of Chile and Brazil

### Characterization of the health system in Chile

The health system in Chile has a mixed financing scheme. First, one source comes from the public system financed with state funds called the National Health Fund (Fonasa). Second, there is a private system with direct financing from families through contracting health plans called Pension Health Institutions (Isapre). Finally, there is a public system of the armed forces (FFAA). According to the National Socioeconomic Characterization Survey [6], 76.5% of the population are enrolled with Fonasa, 15.4% with Isapre, 1.75% to the Armed Forces system, and 4.29% have no affiliation. However, people who are not affiliated are treated free of charge in the public system.

The primary financing source comes from the mandatory health contributions that workers and pensioners provide (7% of the gross income), which are deducted from employers’ remuneration or the pension fund administrator. These will finance the public system (Fonasa) and the private system (Isapres). However, in the case of Fonasa, there is also a contribution from the Government through direct transfers. For its operation until august 2022, Fonasa had a definition of groups so that users can access the benefits. There are four groups according to the individual’s income level. For instance, groups A and B correspond mostly to indigents and migrants, where Fonasa subsidizes or covers 100% of the medical care costs. Also, Fonasa covered 90% and 80% of the medical care costs for groups C and D, respectively. This setup was in rule until July 2022, when the Government announced eliminating all copays starting in September 2022. For Isapre, vouchers and copays vary according to the individual’s contract and the care modality. The modality can be free: the user can choose any public or private healthcare network establishment, or preferential: the user can select a private institution of his preference predefined in the contract. In the private system, the coverage is variable. That is, the private system works like an individual insurance that charges based on the premium paid and the level of risk of the insured (experience rating).

However, individuals can access complementary health insurance to their Isapre or Fonasa plans, which in some cases only have outpatient coverage, coverage associated with high-cost catastrophic diseases, or both. However, as they are financed 100% by the user, only middle- or high-income people access these, so the coverage was 6.0% of the population for the year 2021 [6–8] (Table 1). In this regard, it is important to consider that despite public health policy efforts, catastrophic diseases continue to constitute a significant risk, especially for vulnerable groups [9].

**Table 1:** Indicators of the Chilean Public Health System, 2021. Source: Casen [6], OCDE [7], y Superintendencia de Salud [8]

Indicator	Percentage
Rate of affiliation to the public system (Fonasa)	76.55%
Percentage of the population with supplementary insurance	6.00%
People treated under GES- Explicit Health Guarantees	75.60%
Public expenditure on health as a proportion of total expenditure	49.00%
Health expenditure as a proportion of GDP	8.90%
Nurses per 10,000 inhabitants	4.2
Doctors per 10,000 inhabitants	2.5

The articulators of the health system are the Ministry of Health and the Superintendence of Health. The first is the public body responsible for formulating, adopting, directing, coordinating, executing, and evaluating public policy on health. The second supervises the health system’s performance, promoting and ensuring the equal fulfillment of the people’s health rights.

### Strengths and inefficiencies of the health system in Chile

One of the problems of the public system is that they concentrate on high-risk individuals [9]. This issue puts pressure on the system at specific periods. For example, there is a high prevalence of respiratory diseases during the winter season [10, 11], when the network of public hospitals cannot meet all the demand. The highest prevalence of conditions in the country corresponds to arterial hypertension (7.3% of the population), diabetes (4.36%), and bronchial asthma (1.57%) [6]. In other words, the public system’s shortcomings focus on the problems of attention to users typical of bureaucratic and supply-funded organizational schemes.

In contrast, the private system has the traditional difficulties of individual insurance (risk selection, short-term coverage, and high administrative expenses). Also, to reduce the experience rating of the private system, increase equality and deepen the solidarity principle of the Chilean health system, the Government passed the Law 21,350 of 2021, which regulates the base prices for health plans (premium). Furthermore, this law increased the transparency of the system of private contracts because the Government mandates unique contracts regarding gender and age, which reduced the discrimination of the premium by gender and fertile age and forced the Isapres to accept people with pre-existing diseases.

**Fig. 1:**
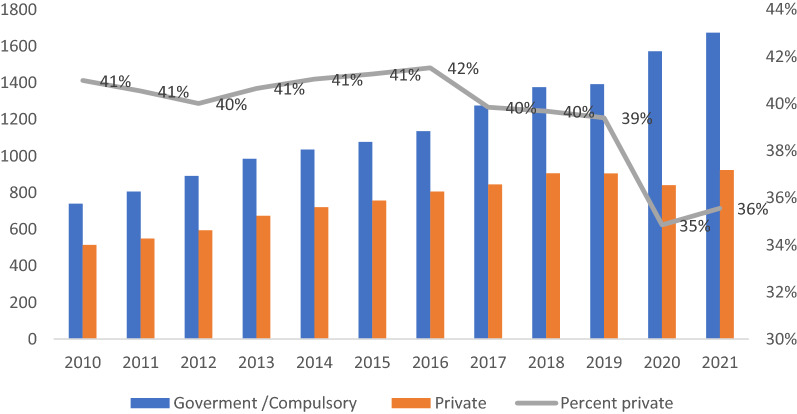
Evolution of public expenditure by source of financing, 2010–2021 (Source: OCDE [20])

Health spending in Chile, public plus private, grew by 91.2% between 2011 and 2021. The primary growth in health spending comes from the public system (Fig. 1), which had to intervene strongly to treat COVID-19 and implement a massive and free vaccination plan in 2020 [12]. More than 74% of the population currently has four doses of vaccines [13]. However, the average private financing in health during the last 10 years has been 40%, demonstrating families’ significant contribution to maintaining the health system.

Additionally, to mitigate the problems of the health system through the formulation of public policies, other measures were implemented, such as the Explicit Health Guarantees (GES) plan. This plan constitutes a set of benefits guaranteed by Law 19,996 of 2005 for people affiliated with Fonasa and Isapres with any of the 85 diseases it covers. This law allows users to be served quickly in any institution of the network of providers. In addition, it limits the maximum time for an individual to be treated. Furthermore, it regulates the private network’s medical attention and hospitalization charges derived from the covered diseases. This plan has comprehensive coverage. For example, 75.6% of the people who have requested it have been able to use it, corresponding to 17.02% of the country’s total population [6]. In addition, the public health system in Chile has important prevention programs. For example, vaccines for vulnerable groups, regardless of income, such as vaccines against influenza, the human papillomavirus, and COVID-19, among others, where people with higher income levels are willing to pay for them [12, 14, 15]. Other issues stressing the health care system periodically is the air pollution generated by industry, vehicle and firewood for heating [11].

Other problems of Chilean health system funding come from the economic perspective: pooling, selection, and risk management. On the one hand, in the public sector, the risk and expected expenditure are inversely proportional to the individual contribution to the health system. This statement is true because individuals with higher prevalence and risks are not admitted or discharged from the private system, ending up enrolled in the public system. That is, the distribution of risk is not equitably. In fact, in the private sector, less than 30% belong to the population is at high risk in health. This phenomenon has led to proposals to modify the health financing system, within which there is a profound shift towards a unique, universal health fund managed by the public sector. That is, the Isapres (private system) would cease to exist. However, this constitutional proposal reform, among others, was rejected in a referendum last September 4, 2022, with 62% of the voting population. Despite the referendum result, a new constitution proposal will be elaborated in the short term, and the idea of a unique health system may appear again. Overall, improving coverage, equity, effectiveness, and efficiency in health provision and financing is challenging, especially considering the country’s demographic transition [16].

Moving people from the private health sector providers (15.4%) to a unique public sector health system will probably stress the current public health system, which has a long waiting list for medical consultation. For example, by March 2022, the public health system did not perform more than 330,000 surgeries. In addition, the average public system waiting time was 603 days, and the waiting list for specialist consultation was 2 million [3]. Therefore, some people, who are now in the private sector, will need to pay additional insurance to maintain quality and timely attention outside of an eventual new public health system under discussion.

### Characterization of the health system in Brazil

The health system in Brazil is mixed, with public and private participation. The public health system is financed mainly by the Government with contributions from the federal, State, and municipal governments, through the Unified Health System (SUS). This system provides the healthcare network and is responsible for coordinating the public system as a whole. It covers 78% of the Brazilian population (Table 2). At the same time, the private system covers only 22% of the people and is called the Supplementary Health System (SS), financed with private individual and corporate funds. This system comprises four types of medical insurance: group medicine, medical cooperatives, self-administered plans by companies, and individual health insurance plans. The SS is implemented by different federal, State, and municipal governments, making the Brazilian public health system to be considered complex [17].

**Table 2:** Brazil: Health system indicators, 2019 (last year available to 2022). Source: OCDE [21]

Indicator	Percentage
Rate of affiliation to the Unified Health System (SUS)	78%
Public expenditure on health per capita	853 USD
Health expenditure as a proportion of GDP	9.6%
Nurses per 10,000 inhabitants	8
Doctors per 1000 inhabitants	2.3

### Strengths and inefficiencies of the health system in Brazil

One of the most effective plans to improve public health, through primary care, of the vulnerable population has been the Family Health Strategy (FHS). This strategy aims at reorganizing (expansion, qualification, and consolidation) primary care in the country [18]. However, according to the analysis carried out by Harris and Libardi Maia [19], the private sector has grown over time, generating influence in health policy and weakening the public sector, despite the growing efforts of the latter. Regarding the organizational structure of the health system, the body responsible for ensuring quality and regulating private health insurance is the National Council of Supplementary Health (ANS). The National Health Surveillance Agency (ANVISA) is responsible for health promotion and prevention.

**Fig. 2:**
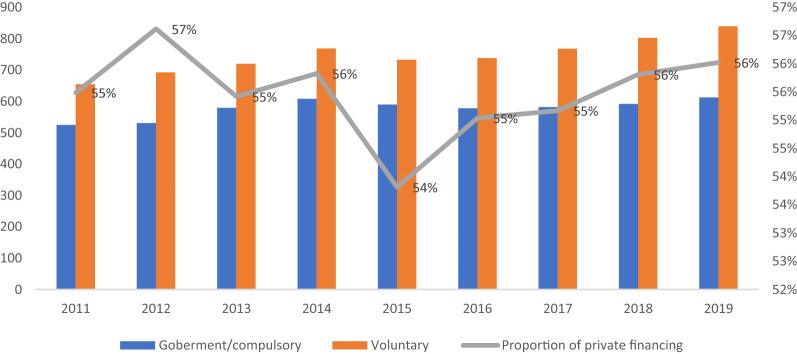
Brazil: Health spending by funding source, 2011–2019 (Source: OCDE [20])

Brazil was the third OECD country in Latin America with the lowest per capita health expenditure by 2021 [20]. However, it spends 9.6% of its GDP on health, which is higher than the average of OECD countries, corresponding to 8.8% for 2021 [21]. Additionally, there is a growing private health financing, reaching 56% of total health spending in 2019 (Fig. 2). This low per capita expenditure is explained by the vast population that needs to be financed: Brazil is the most populous country in the Americas and the fourth worldwide.

Brazil’s public health system’s weaknesses were evidenced by the COVID-19 pandemic, especially governance-related [22], mainly due to the structural problem of the efficiency of hospital care in the public system, financing problems, and lack of human resources for management [23]. Furthermore, according to OECD (2021), there was a lack of clarity in the communication strategy for COVID-19 and misaligned practical measures. This is explained by the constant changes of authorities in the Ministry of Health. Also, there were infrastructure shortages to face the pandemic. In fact, as of July 24, 2022, 62.5% of the population is fully vaccinated against COVID-19 (2 doses) [24]. However, there are countries in Latin America with higher vaccination rates, as is the case of Chile, with 92.2% of its population with four doses. These percentages demonstrate the challenges of a large population and space country like Brazil.

The main problems facing the public health system are the demographic transition and weaknesses in the effectiveness and efficiency of public health provision. Those problems have been deepened by the epidemiological transition and the COVID-19 pandemic [25]. For this reason, Brazil needs to improve the scope and coverage of health in the regions, especially those that need to modernize primary care services, healthcare centers, and hospitals. In addition, there is a lack of coordination between primary and secondary care, and there is an irregular distribution of integrated care models throughout Brazil. The solution to those issues requires increasing the financing of the SUS. Succinctly, the public health system in Brazil needs to improve the offer of services, benefits, and infrastructure to offer a more equitable, quality, and financially sustainable SUS.

## Comparison of the health system and health expenditure of Chile and Brazil

The current health systems of Chile and Brazil are in some way different because Brazil has a Universal Health System, and Chile has a dual Health System. However, there are some proposals from some political parties in Chile to move to a Unified Health System, which is presently in discussion to be included in a possible new Chilean Constitution.

The literature recognizes multiple weaknesses in the public health system in Latin America, where several are presented in the Brazilian and Chilean public health systems. Gomez-Temporao and Faria [5] mention, for example, the segmentation of the health system, inadequate supply of primary services, lack of capacity of the Health Secretariat of Government to obtain new resources, the deficit of human resources and specialists, extended times for decision-making to build new facilities or hospitals, and inequality in coverage. In addition, especially in the case of Brazil, because of the its large population and geography extension and access, there is reduced health coverage in some areas. While in the Chilean case, the access to remote areas is easier because it has very good connectivity along the territory.

In recent years, more significant social movements have been calling on governments to prioritize and guarantee better health, which has improved coverage and some services. Additionally, health prevention policies have been strongly promoted to reduce the number of people who come to health centers for help. These kinds of programs are significant in Chile. Another related issue is the high price of medicines and their quasi-monopolistic supply position. Like other countries, this limits life-saving medication access to the poorest people [26].

Castro [27] reports that the unified health system has allowed Brazil to address the population’s health needs. However, at the same time, she recognized that the system needs to consider some upgrades to reduce regional inequalities, increase funding resources, and increment private sector–public sector collaboration.

De acuerdo al OECD [28], resume some highlights about the Brazil health system: (a) Health spending in Brazil should improve efficiency in primary health care, hospital care, pharmaceuticals, long-term care and governance; (b) it require to create a long-term care services for older population wich is increasing; pharmaceutical spending is pay mainly by out-of-pocket payments, affecting more to poor people. These issues also are relevant for the Chilean case. As several other countries, the healthcare systems are challenged by population aging, innovation in medical technology and increasing demand for healthcare services [29].

**Fig. 3:**
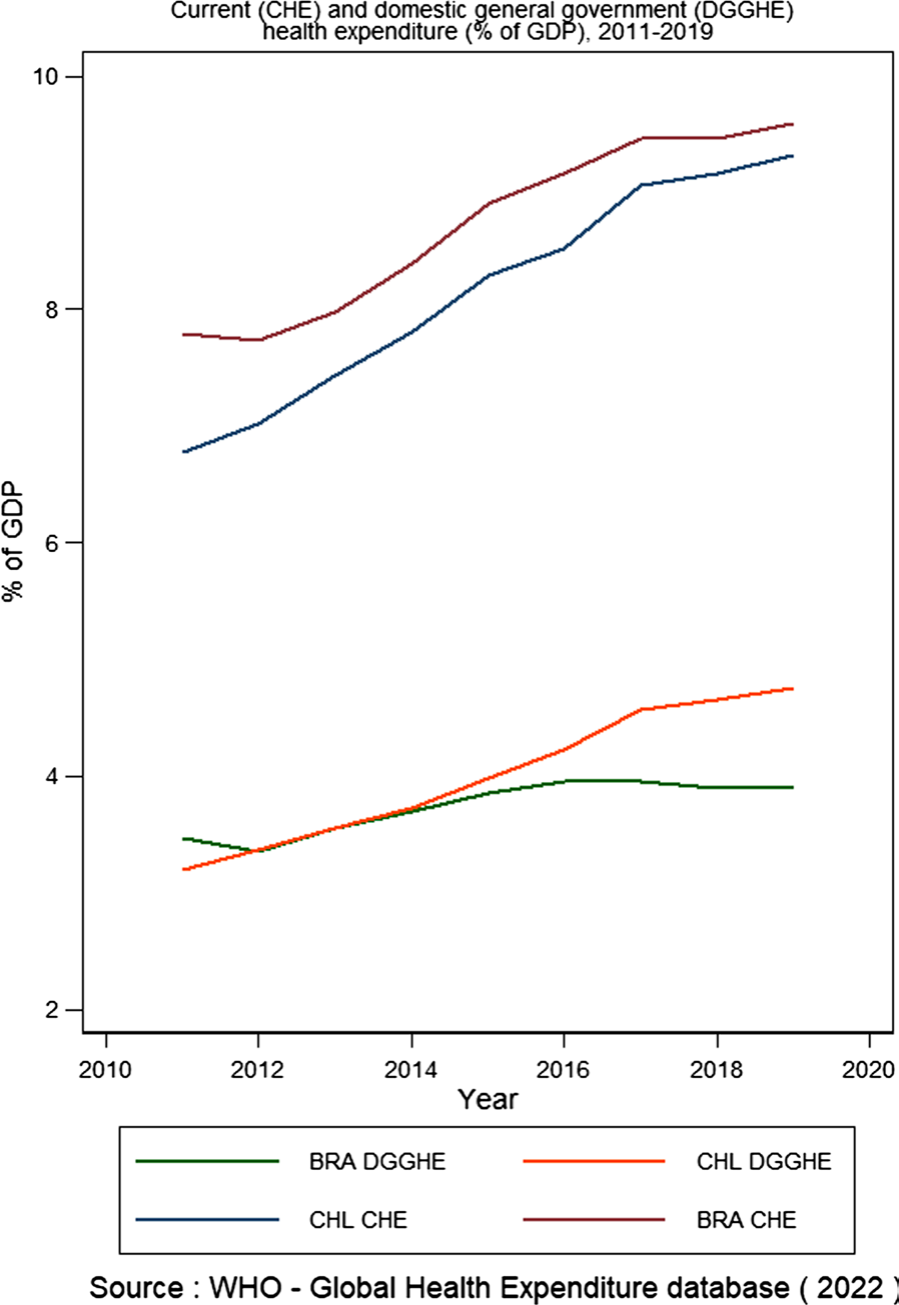
Comparison of Brazil (BRA) and Chile’s (CHL) current and domestic general government health expenditure as % of GDP, 2011–2019

In the last decade, Brazil and Chile raised health expenditure from 7.8 to 9.6% of the GDP and 6.8 to 9.3% of the GDP, respectively. Figure 3 shows that current health expenditures have increased more rapidly in Chile than in Brazil, despite the implementation of the universal health system in Brazil. Moreover, if the Government’s domestic expenditure on health is compared, it is also observed that growth has been more relevant in Chile, going from 3.1 to 4.75%. In contrast, Brazil grew from 3.5 to 3.9% of the GDP.

**Fig. 4:**
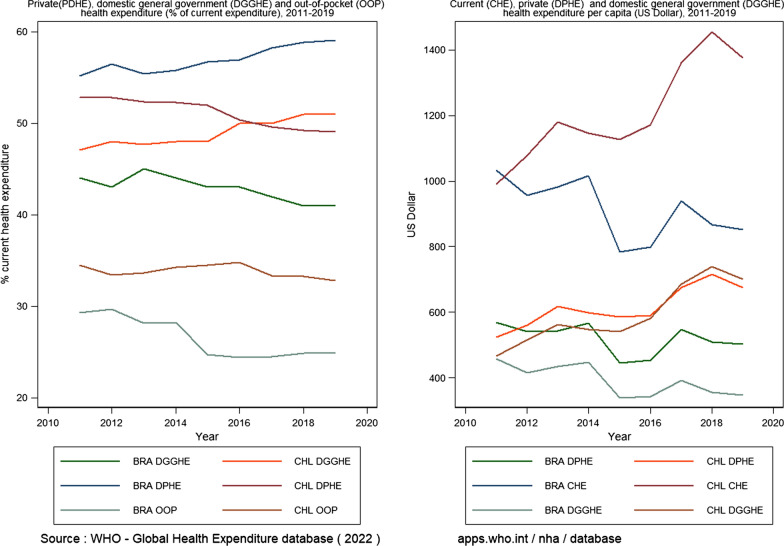
Comparison of Brazil (BRA) and Chile (CL) health expenditure by different sources as % of current spending and in per capita US Dollar, 2011–2019

Figure 4 compares the different sources of health expenditure per capita between Brazil and Chile. The out-of-pocket health expenditure (OOPHE) has been reduced in both countries but more significantly in Brazil. However, the private domestic health expenditure (PDHE) has increased in Brazil but decreased in Chile. This is undoubtedly the result of the increase in government spending on health (DGGHE) in Brazil, which is accompanied by a higher increase in the population. Additionally, as Castro [27] mentioned, this results from restrictive fiscal policies implemented in 2016, intending to reduce expenditure in the country, as can be observed in Fig. 4. Most countries depend on economic growth to continue improving healthcare services.

## Conclusions

The health systems in Brazil and Chile are different. Brazil has a unique health system, and Chile has a dual health system. The growth in the per capita current health expenditure in Chile has been higher than in Brazil, but both countries still need to work significantly to improve access and speed of healthcare for the neediest people. Brazil has more limitations in covering health care services because of more complicated geographic characteristics and a larger size than Chile. Additionally, a better institutional framework should be given for the participation of the private health sector. For instance, in Chile, the Supreme Court limits the increase in the prices of private health plans, which is affecting the sector’s viability. Additionally, the process of building new hospitals or health centers should be improved because allocations and constructions are too slow due to legal and administrative reasons.

## Data Availability

The extracted data from the systematic review are available from the corresponding author upon reasonable request.

## Abbreviations

OECD/OCDE: The Organisation for Economic Co-operation and Development
CASEN: Survey—National Socioeconomic Characterization
WHO: World Health Organization
SUS: Unique Health System
